# 4440 Enhanced radiation therapy using chlorin-e6 conjugated gold nanoparticles

**DOI:** 10.1017/cts.2020.330

**Published:** 2020-07-29

**Authors:** Samir V Jenkins, Robert J. Griffin

**Affiliations:** 1 University of Arkansas Translational Research Institute; 2 UAMS

## Abstract

OBJECTIVES/GOALS:

- Development of gold nanoparticles covalently linked to a photosensitizer for use to enhance radiation therapy. The particles will be thoroughly characterized structurally and mechanistically. The gold particles should enhance radiation activity by closer proximity to the photosensitizer and by increasing particle accumulation in the tumor.

METHODS/STUDY POPULATION:

- Gold nanoparticles were synthesized and coated with amine-terminated poly(ethylene) glycol, then covalently conjugated to chlorin e6, a known FDA-approved photosensitizer. The system was characterized using UV-Vis spectroscopy, transmission electron microscopy, and nanoparticle tracking analysis. The generation of reactive oxygen species was measured after X-irradiation. Enhanced cell killing was evaluated clonogenically in addition to assessment of *in vivo* efficacy and tumor pathology.

RESULTS/ANTICIPATED RESULTS:

- Conjugation of the particle to the photosensitizer was achieved, and the molecule was detected by UV-Vis spectroscopy. TEM and NTA showed no aggregation of the particles, and an increase in reactive oxygen species generation was observed. The conjugates increased cell killing during radiation treatment, whereas neither the particle alone nor the photosensitizer significantly affected clonogenic survival at the same concentrations. Breast tumors grown in immunocompetent mice showed increased necrotic tissue after a single 20 gy treatment in the presence of the conjugate.

DISCUSSION/SIGNIFICANCE OF IMPACT: Radiation therapy is widely used clinically, but dosage is limited largely to prevent injury to adjacent normal tissue. By increasing the local effect of radiation therapy, our gold conjugate has the potential to augment the effective radiation dose in the tumor, thereby reducing damage to healthy tissue and providing a more effective therapy.

